# Stronger selection can slow down evolution driven by recombination on a smooth fitness landscape

**DOI:** 10.1371/journal.pone.0183120

**Published:** 2017-08-15

**Authors:** Masahiko Ueda, Nobuto Takeuchi, Kunihiko Kaneko

**Affiliations:** 1 Department of Basic Science, The University of Tokyo, Komaba, Meguro-ku, Tokyo 153-8902, Japan; 2 Research Center for Complex Systems Biology, The University of Tokyo, Komaba, Meguro-ku, Tokyo 153-8902, Japan; Centrum Wiskunde & Informatica (CWI) & Netherlands Institute for Systems Biology, NETHERLANDS

## Abstract

Stronger selection implies faster evolution—that is, the greater the force, the faster the change. This apparently self-evident proposition, however, is derived under the assumption that genetic variation within a population is primarily supplied by mutation (i.e. mutation-driven evolution). Here, we show that this proposition does not actually hold for recombination-driven evolution, i.e. evolution in which genetic variation is primarily created by recombination rather than mutation. By numerically investigating population genetics models of recombination, migration and selection, we demonstrate that stronger selection can slow down evolution on a perfectly smooth fitness landscape. Through simple analytical calculation, this apparently counter-intuitive result is shown to stem from two opposing effects of natural selection on the rate of evolution. On the one hand, natural selection tends to increase the rate of evolution by increasing the fixation probability of fitter genotypes. On the other hand, natural selection tends to decrease the rate of evolution by decreasing the chance of recombination between immigrants and resident individuals. As a consequence of these opposing effects, there is a finite selection pressure maximizing the rate of evolution. Hence, stronger selection can imply slower evolution if genetic variation is primarily supplied by recombination.

## Introduction

It is commonly expected that the rate of evolution is higher when selection is stronger [1]. This is because stronger selection ensures fitter genotypes created by mutation to survive. Indeed, it is well known that in a weak mutation regime (i.e. for sufficiently low mutation rates) and on a smooth fitness landscape [2, 3], the rate of evolution *v* is described as

$$\begin{array}{ccc} v & = & 4Nus, \end{array}$$ (1)

where *N* is the population size, *u* is the beneficial mutation rate, and *s* is the selection coefficient [4]. This equation shows that the rate of evolution *v* increases linearly with the strength of selection *s*. Such monotonic dependence of *v* on *s* is expected to persist even in a strong mutation regime. In this regime, beneficial mutations can arise simultaneously and interfere with each other’s fixation, a phenomenon known as clonal interference. Although clonal interference decreases *v*, making it less than proportional to *N* and *u* (cf. Eq 1), *v* still increases monotonically with *s* [5–9]. Therefore, there is no finite value of selection pressure maximizing the speed of evolution.

However, this monotonic dependence of *v* on *s* has been derived under the assumption that genetic variation within a population is primarily created by mutation (mutation-driven evolution, for short). In this paper, we show that this widely-known relationship does not actually hold if genetic variation within a population is primarily created by migration and recombination (recombination-driven evolution, for short), even for a smooth fitness landscape. Recombination is a source of new genotypes besides mutation. Recombination between genomes occurs in sexual reproduction and is beneficial in avoiding Muller’s ratchet and clonal interference [10–12]. Furthermore, a type of recombination known as horizontal gene transfer is considered to be important also in the evolution of prokaryotes [13–18].

Specifically, we consider a situation in which novel genes are supplied to a population through immigration from other populations followed by recombination between migrant and resident individuals (i.e. introgression). To ensure the generality of results, we investigate two models representing distinct evolutionary scenarios. The first model considers migration and recombination between populations adapting to multiple distinct ecological niches (Model 1). The second model considers migration and recombination between populations adapting to a single common ecological niche (Model 2). Under both the models, we find that there is an optimal selection pressure maximizing the speed of evolution; i.e., *v* is a non-monotonic function of *s*.

## Model 1

We assume that there are many populations, each of which is evolving toward adaptation to a distinct ecological niche. A population occasionally receives immigrants from the other populations. The immigrants always have fitness lower than that of resident individuals owing to differences in niches. However, the genomes of the immigrants are assumed to contain genes that are beneficial to the resident individuals, but are absent in the latter. These genes can be transferred to the latter through recombination. For simplicity, only the dynamics of a single population is explicitly considered, with that of the others abstracted away on the basis of the mean-field-like approximation as described below.

Throughout the paper, mutation is assumed to be rare enough to be negligible in order to focus on recombination-driven evolution. The fitness landscape is assumed to be smooth so that the fitness landscape in itself does not cause the non-monotonic dependence of the speed of evolution on selection pressure (see also Discussion).

### Methods

Model 1 assumes a population of *N* individuals (see Table 1 for notation). The genotype of individual *i* ∈ {1, ⋯, *N*} is denoted by ***g***_*i*_ ≡ (*g*_1, *i*_, ⋯, *g*_*L*, *i*_). Each variable *g*_*l*, *i*_ denotes a type of a gene (i.e. allele) at locus *l* ∈ {1, ⋯, *L*} and assumes either the value of −1 (deleterious) or 1 (beneficial) [19].

**Table 1:** List of symbols.

*N*	population size
*L*	the number of gene loci
*r*	recombination rate per individual per loci
*μ*	migration rate for Model 1
*D*	migration rate for Model 2
*N*_*s*_	the number of subpopulations in Model 2
*s*	selection pressure
*g*_*l*_	gene at locus *l* (*g*_*l*_ ∈ {−1, 1})
***g***	genotype defined as (*g*_1_, ⋯, *g*_*L*_)
*ϕ*(***g***)	rescaled fitness defined as ∑_*l*_ *g*_*l*_ (fitness is defined as *e*^*sϕ*(***g***)^)
*ϕ*_0_	difference in rescaled fitness between resident individuals and migrants
*v*	the rate of evolution defined as 〈*ϕ*〉 = *vt* + *const*.

We consider the time evolution of the system, which consists of three discrete steps: selection, recombination and migration. In the selection step, *N* genotypes are selected from the present population with probabilities proportional to the fitness of genotypes. The fitness of genotype ***g*** is defined as exp(*sϕ*(***g***)), where *ϕ*(***g***) is rescaled fitness defined as

$$\begin{array}{c} \phi (g)\equiv \sum_{l=1}^{L}g_{l} \end{array}$$ (2)

((*ϕ* + *L*)/2 counts the number of beneficial alleles in a genome), and *s* is selection pressure. Accordingly, the probability that individual *j* is selected for reproduction is

$$\begin{array}{ccc} P(j) & = & \frac{e^{s\phi (g_{j})}}{\sum_{i=1}^{N}e^{s\phi (g_{i})}}. \end{array}$$ (3)

Note that if *ϕ*(***g***_*j*_) > *ϕ*(***g***_*k*_), *P*(*j*)/*P*(*k*) increases with *s*; thus, the larger the value of *s*, the stronger natural selection. Note also that the fitness landscape is smooth because it contains only one local and global maximum and one local and global minimum.

In the recombination step, individuals exchange genes with probability *r* per individual per locus per generation:

$$\begin{array}{ccc} & & \left(\cdots ,g_{l-1,i},g_{l,i},g_{l+1,i},\cdots )+(\cdots ,g_{l-1,j},g_{l,j},g_{l+1,j},\cdots \right)\\ & \to & (\cdots ,g_{l-1,i},g_{l,j},g_{l+1,i},\cdots )+(\cdots ,g_{l-1,j},g_{l,i},g_{l+1,j},\cdots ). \end{array}$$ (4)

Pairs of individuals undergoing recombination are selected randomly.

Migration occurs from the other populations (pool) to the system. We assume that individuals change with probability *μ* as

$$\begin{array}{ccc} g_{i} & \to & g^{\left(\text{pool}\right)}, \end{array}$$ (5)

where genotype ***g***^(pool)^ is randomly generated with rescaled fitness *ϕ*(***g***^(pool)^) = *ϕ*(***g***_*i*_) − *ϕ*_0_ with *ϕ*_0_ > 0. That is, migration always decreases the fitness of the system, but sequences ***g***^(pool)^ and ***g***_*i*_ are uncorrelated (for this reason, the effect of migration differs from that of introducing *ϕ*_0_/2 deleterious mutations). The rescaled fitness difference *ϕ*_0_ between a resident individual and a migrant is set constant under the assumption that individuals in the other populations also evolve at the same rate as those in the focal population.

For each simulation, the model was initialized with individuals having random genotypes and rescaled fitness *ϕ* = 0. The parameters were set as follows: *N* = 1000 or 2000, *L* = 1000, *r* = 10^−4^ or 2 × 10^−4^, *μ* = 10^−3^ or 2 × 10^−3^, and *ϕ*_0_ = 20. Statistical quantities were calculated by running 1000 replicate simulations.

### Results

**Fig 1:**
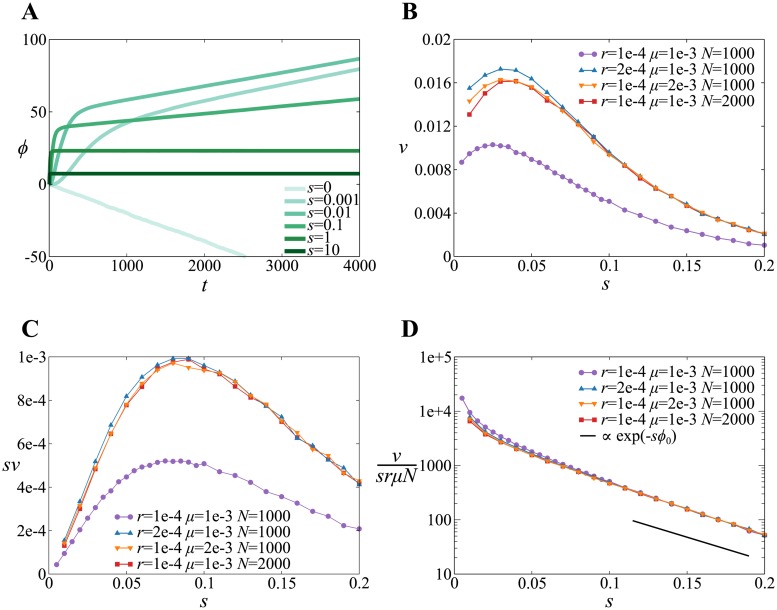
**A**. The average rescaled fitness 〈*ϕ*〉 as a function of time *t* for various strengths of selection (denoted by *s*) for Model 1. *ϕ* is proportional to the number of beneficial alleles in a genome (which is (*ϕ* + *L*)/2). The parameters are as follows: *N* = 1000, *L* = 1000, *r* = 10^−4^, *μ* = 10^−3^, and *ϕ*_0_ = 20 (see Table 1 for notation). **B**. The rate of evolution Δ*ϕ*/Δ*t* (denoted by *v*) as a function of *s* for Model 1. *L* = 1000 and *ϕ*_0_ = 20 (the other parameters are indicated in the graph). **C**. The rate of evolution in Malthusian fitness (i.e. a logarithm of fitness) *sv* as a function of *s* for Model 1. The parameters are the same as in B. **D**. A semi-log plot of *v*/(*srμN*). The parameters are the same as in B. The slope of the solid line is −*ϕ*_0_.

We numerically calculated the time evolution of the average rescaled fitness 〈*ϕ*〉, where 〈⋯〉 denotes a population average. The result indicates that the dynamics of 〈*ϕ*〉 has two phases as described below (Fig 1A).

In the first phase, 〈*ϕ*〉 rapidly increases in a sigmoidal manner, except for *s* = 0 (Fig 1A, *t* < 1/*s*). During this phase, the effect of migration is negligible because the migration rate *μ* is set to a value smaller than or equal to *s* (specifically, *μ* = 10^−3^). Thus, this phase constitutes an initial, transient dynamics before migration takes effect, resulting from selection and recombination within a population. During this phase, the system becomes increasingly homogeneous as selection removes genetic variations with virtually no supply of new genes through migration. Eventually, one genotype is selected, whose fitness depends on the selection pressure and the recombination rate. Since the frequency of the fittest genotype increases exponentially as *e*^*st*^, the first phase lasts until *e*^*st*^ ∼ 1. Therefore, the duration of the first phase scales approximately as 1/*s*.

In the second phase, 〈*ϕ*〉 increases almost linearly at a rate that depends on the value of *s* (Fig 1A, *t* > 1/*s*). In this phase, a quasi-steady state is achieved, in which a population is almost homogeneous, but continually receives immigrants and incorporates new genes supplied by them through recombination and selection. Thus, this phase constitutes evolution driven by recombination and migration. Note, however, that 〈*ϕ*〉 eventually saturates on an even longer time scale (*t* ≫ 4000) as a trivial consequence of the fact that *ϕ* has the maximum value *L*. Since we are interested in the rate of evolution driven by recombination and migration, we hereafter focus on the second phase of the dynamics well before this saturation occurs.

Note a special case arising for *s* = 0, for which 〈*ϕ*〉 decreases monotonically (Fig 1A). This decrease is due to the assumption that immigrants always have fitness lower than that of residents owing to differences in niches—the assumption that becomes senseless when *s* = 0. We do not consider this special case hereafter because we are interested in the evolution under selective pressure and in testing the monotonic dependence of the rate of evolution on *s* as implied by Eq (1), which is derived under the assumption that *Ns* ≫ 1.

Fig 1A suggests that the rate at which 〈*ϕ*〉 increases at *t* ≃ 4000 displays non-monotonic dependence on *s*. In particular, the rate of evolution in 〈*ϕ*〉 seems to be maximized at *s* ≃ 10^−2^. To confirm this result, we next computed the slope of 〈*ϕ*〉 (denoted by *v*) as a function of *s*. The value of *v* was obtained by fitting a linear equation *vt* + *C* to the curve of 〈*ϕ*〉 in the range of *t* ∈ [2000, 4000] by the least squares method. The result shows that *v* depends non-monotonically on *s* (Fig 1B), indicating that evolution slows down as selection pressure increases, even though the fitness landscape is smooth. The value of selection pressure maximizing the rate of evolution is approximately 0.025 for the parameters used in Fig 1.

The existence of a finite selection pressure maximizing the rate of evolution *v* stems from the two opposing effects of natural selection on *v*. On the one hand, selection increases the fixation probability of fitter genotypes, hence positively contributing to *v*. One the other hand, selection decreases the residence time of immigrants, negatively contributing to *v*, as described below. The genotypes of immigrants are uncorrelated with those of the individuals already present in a population. Thus, the immigrants can provide beneficial genes to the population if they survive selection and recombine with the resident individuals. However, the survival of the immigrants is hampered by selection because their fitness is smaller than that of the resident individuals. The duration for which the immigrants survive (the residence time, for short) decreases with selection pressure. Therefore, the probability that the population obtains beneficial genes through recombination decreases as selection pressure increases. Owing to these two opposing effects, there is a finite selection pressure maximizing the rate of evolution.

The above intuitive argument can be made more quantitative by estimating *v* as a function of *s* as follows. The value of *v* is approximately proportional to the rate at which novel beneficial genes are supplied to a population (for simplicity, we ignore clonal interference between resident individuals independently gaining beneficial genes from migrants via recombination; this simplification is not expected to affect our conclusion as described later). Such genes are supplied through immigration followed by recombination between immigrants and resident individuals. Thus, the rate of this supply is proportional to *μ* (migration rate), *r* (recombination rate), *N* (population size), *L* (the number of loci), the residence time of migrants (denoted by *τ*(*s*)), and the probability that an immigrant carries a novel beneficial gene per locus (denoted by *ρ*). Furthermore, the fixation of a beneficial gene occurs with a probability proportional to *s* for *s* ≪ 1 and *Ns* ≫ 1 [4]. Therefore, *v* is estimated as

$$\begin{array}{ccc} v & \propto & N\rho rL\mu \tau (s)s \end{array}$$ (6)

for *N*^−1^ ≪ *s* ≪ 1. Eq (6) differs from Eq (1), in that the former contains *τ*(*s*), a factor that negatively depends on *s*, whereas the latter contains no such factor.

The probability *ρ* generally depends on the fitness *ϕ*; however, *ρ* can be regarded as constant in our simulations. Suppose that the average number of beneficial genes in the genomes of resident individuals is *l*. Then, a migrant has *l* − *ϕ*_0_/2 beneficial genes. Recombination succeeds in increasing fitness only if a deleterious gene of a resident individual is exchanged with a beneficial gene of a migrant—this occurs with the probability *ρ* = (*L* − *l*)/*L* × (*l* − *ϕ*_0_/2)/*L*. This probability *ρ* takes the maximum at *l* = (*L* + *ϕ*_0_/2)/2 ≃ *L*/2. Because we only consider a time range within which *l* ≃ *L*/2 (more precisely, 500 ≤ *l* < 550 during any simulation), *ρ* can be regarded as nearly constant.

The residence time *τ*(*s*) can be roughly estimated as *e*^−*ϕ*_0_*s*^, as follows. First, we consider the situation in which one migrant with fitness *e*^*s*(*ϕ*−*ϕ*_0_)^ migrates into a population of (*N* − 1) individuals with fitness *e*^*sϕ*^. The probability that the migrant dies out in the next selection step is calculated from Eq (3) as

$$\begin{array}{c} d={\left\{\frac{\left(N-1\right)e^{s\phi }}{\left(N-1\right)e^{s\phi }+e^{s\left(\phi -\phi_{0}\right)}}\right\}}^{N}≃\text{exp}\left(-e^{-s\phi_{0}}\right), \end{array}$$ (7)

where we have used the fact that *N* ≫ 1. When we write the probability distribution of residence time as *p*(*t*) = (1 − *d*)^*t*^ *d*, the average residence time is calculated as

$$\begin{array}{ccc} \left\langle t\right\rangle & = & \sum_{t=0}^{\infty }tp\left(t\right)=\frac{1-d}{d}. \end{array}$$ (8)

By using the above expression for *d*, we finally obtain

$$\begin{array}{ccc} \tau (s) & = & \langle t\rangle =\exp(e^{-s\phi_{0}})-1≃e^{-s\phi_{0}} \end{array}$$ (9)

for *sϕ*_0_ ≫ 1.

Taken together, the above results indicate that

$$\begin{array}{ccc} v & \propto & Nr\mu e^{-s\phi_{0}}s \end{array}$$ (10)

for large values of $s\gg \phi_{0}^{-1}$. Therefore, *v* decreases exponentially for large *s*, whereas it increases linearly for small *s*, with a crossover around s_*_ ≃ 1/*ϕ*_0_—i.e. *v* depends on *s* non-monotonically. Eq 10 also implies that *v* is proportional to *Nrμ*. This implication is supported by Fig 1B, which shows that the values of *v* for *Nrμ* = 2 × 10^−4^ collapse into the same curve for different values of *N*, *r* and *μ*, and that these values are almost twice the values of *v* for *Nrμ* = 10^−4^, provided *s* > 0.1. Moreover, a semi-log plot of *v*/(*Nrμs*) for various values of *N*, *r*, and *μ* shows that all data points collapse into a single line with a slope close to −*ϕ*_0_ for *s* > 0.1, as predicted by Eq 10 (Fig 1D). Taken together, these results support the validity of Eq (10).

The derivation of Eq (10) neglects clonal interference between resident individuals that independently gain beneficial genes from migrants. This simplification is unlikely to affect the conclusion that *v* depends on *s* non-monotonically for the following reason. The effect of clonal interference, which is always to decrease *v*, is expected to diminish as *s* increases, because the residence time of a migrant decreases exponentially with *s* according to Eq (9). This expectation implies that allowing for clonal interference would not alter non-monotonic dependence on *s* itself but only shift the location of the maximum of *v* in Eq (10) along the *s* axis. In addition, the above expectation implies that clonal interference diminishes the precision of Eq (10) for small values of *s*, an implication that might explain the dispersion of data points for *s* < 0.1 in Fig 1D.

The rate of evolution *v* considered above is defined in terms of changes in genotypes because *v* is calculated from rescaled fitness *ϕ*. Alternatively, the rate of evolution can also be defined in terms of changes in fitness. In this case, *sv* rather than *v* should be considered because *sv* is the rate of the change of *sϕ*, which is a logarithm of fitness (i.e. Malthusian fitness). Under this definition, there is still a finite selection pressure maximizing the rate of evolution (Fig 1C). Therefore, evolution can slow down as selection pressure increases both in genotype space and in fitness space (however, the latter result does not hold for Model 2 as described below).

## Model 2

To investigate the generality of the results obtained with Model 1, we next consider a situation in which multiple spatially-separate subpopulations are evolving toward adaptation to a common ecological niche. A subpopulation occasionally receives immigrants from the other subpopulations. Since the subpopulations share the same niche, the fitness of immigrants can be higher than that of resident individuals depending on the degrees to which different subpopulations have adapted to the niche. This situation may correspond to Model 1 with *ϕ*_0_ fluctuating around 0. However, because determining the distribution of *ϕ*_0_ is difficult, here we explicitly consider multiple subpopulations. As in Model 1, mutation is ignored to focus on recombination-driven evolution.

### Methods

We consider a population consisting of *N*_*s*_ subpopulations. Each subpopulation contains *N* individuals. The genotype of individual *i* ∈ {1, ⋯, *N*} in subpopulation *a* ∈ {1, ⋯, *N*_*s*_} is denoted by $g_{i}^{\left(a\right)}\equiv \left(g_{1,i}^{\left(a\right)},\cdots ,g_{L,i}^{\left(a\right)}\right)$. Each variable $g_{l,i}^{\left(a\right)}$ takes the value −1 (deleterious) or 1 (beneficial) as before.

The time evolution of the system consists of three steps as in Model 1: selection, recombination, and migration. The selection step in each subpopulation is the same as in Model 1 (see Eqs (2) and (3)). In the recombination step, individuals in the same subpopulation exchange genes. The exchange

$$\begin{array}{ccc} & & \left(\cdots ,g_{l-1,i}^{\left(a\right)},g_{l,i}^{\left(a\right)},g_{l+1,i}^{\left(a\right)},\cdots )+(\cdots ,g_{l-1,j}^{\left(a\right)},g_{l,j}^{\left(a\right)},g_{l+1,j}^{\left(a\right)},\cdots \right)\\ & \to & \left(\cdots ,g_{l-1,i}^{\left(a\right)},g_{l,j}^{\left(a\right)},g_{l+1,i}^{\left(a\right)},\cdots )+(\cdots ,g_{l-1,j}^{\left(a\right)},g_{l,i}^{\left(a\right)},g_{l+1,j}^{\left(a\right)},\cdots \right) \end{array}$$ (11)

occurs with probability *r* per individual per locus per generation. Pairs of individuals undergoing recombination are selected randomly.

In the migration step, individuals migrate between subpopulations. Individuals change as

$$\begin{array}{ccc} g_{i}^{\left(a\right)} & \to & g_{j}^{\left(b\right)} \end{array}$$ (12)

for each pair of individuals (*i*, *j*) and for each pair of subpopulations (*a*, *b*) with probability *D* (spatial structure is ignored). That is, individual *i* in subpopulation *a* is replaced by a copy of the individual *j* in subpopulation *b*.

We set initial conditions as random configurations with rescaled fitness *ϕ* = 0 for each genotype $g_{i}^{\left(a\right)}$. Parameters were set as *N* = 1000, *N*_*s*_ = 64, *L* = 1000, *r* = 10^−4^, and *D* = 10^−7^/64^2^. Statistical quantities were calculated by running 1000 replicate simulations.

### Results

**Fig 2:**
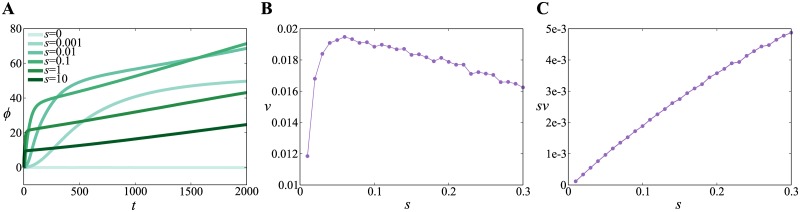
**A**. The average rescaled fitness 〈*ϕ*〉 as a function of time *t* for various strengths of selection (denoted by *s*) for Model 2. *ϕ* is proportional to the number of beneficial alleles in a genome (see Table 1 for details). **B**. The rate of evolution Δ*ϕ*/Δ*t* (denoted by *v*) as a function of *s* for Model 2. **C**. The rate of evolution in Malthusian fitness (i.e. a logarithm of fitness) *sv* as a function of *s* for Model 2.

We display the time evolution of the average rescaled fitness 〈*ϕ*〉 in Fig 2A. We find that the dynamics of 〈*ϕ*〉 consists of the two phases, as in Model 1. In addition to saturation due to the finiteness of *L*, it should be noted that 〈*ϕ*〉 saturates for *t* ≫ 1000 because genotypes of all subpopulations eventually become homogeneous in recombination-driven evolution. However, this saturation is expected to disappear as *N*_*s*_ increases to infinity, and we focus on the second phase driven by both recombination and migration. The slope of the linear parts in *t* ≃ 2000 has non-monotonic *s* dependence, a result that is the same as in Model 1. The slope *v* of this linear region is also estimated by fitting of the *t* ∈ [1000, 2000] part of the curves, and is plotted for various *s* in Fig 2B. We observe the existence of the finite selection pressure value maximizing the rate of evolution at *s* ≃ 0.06.

The mechanism of this phenomenon is explained as follows. In the second phase, a quasi-steady state is realized and dominant process is migration. When an individual migrates into a subpopulation, the migrant and other individuals in the subpopulation can incorporate the beneficial genes of each other by recombination. This process most likely succeeds in intermediate selection pressure *s*. For small *s*, selection does not work effectively. For large *s*, the subpopulation does not have enough time to take in the information provided by the migrant. Therefore, there exists finite selection pressure maximizing the rate of evolution.

For Model 1, we found that the selection pressure maximizing the velocity in fitness space *sv* is also finite as displayed in Fig 1C. In contrast, we find that a selection pressure maximizing *sv* is infinite for Model 2, as displayed in Fig 2C. We note that Eq (6) is also expected to hold for Model 2. We therefore consider that the difference between the two models comes from the difference in the meaning of *τ*(*s*). In Model 1, migration always decreases the fitness by definition, and *τ*(*s*) is the residence time of the migrant before dying out. In Model 2, migration can both increase and decrease the fitness of a subpopulation, and *τ*(*s*) is the time during which the migrant stays in the subpopulation. Since migrants with higher fitness do not necessarily die out in Model 2, *τ*(*s*) does not decrease exponentially for large *s*, but decreases much slowly. This slower decrease in *τ*(*s*) is likely to be the reason why *sv* does not have a maximum at finite *s*, although *v* has. Further investigation will be needed for this topic.

## Discussion

In this paper, we investigated evolution driven by selection, recombination and migration on a smooth fitness landscape. We find that the speed of evolution can slow down as selection pressure *s* increases without the ruggedness of the fitness landscape *ϕ*(***g***). Our results suggest that an optimal selection pressure exists for evolution driven by recombination, in contrast to evolution driven by mutation.

Before ending this paper, we make six remarks. The first remark is related to the inclusion of the effect of mutation. We conjecture that whether mutation or recombination is dominant is determined by comparing two expressions (1) and (6). The selection pressure maximizing the rate of evolution will become infinity in the presence of frequent mutation in addition to recombination, while our recombination-driven result will be reproduced in a weak mutation regime. At the simplest level, we estimate that mutation-driven situation is realized when *u* ≫ *rμτ*(*s*), while recombination-driven situation is realized when *u* ≪ *rμτ*(*s*). The verification of this conjecture and analysis of the intermediate regime *u* ≃ *rμτ*(*s*) are one of the future problems.

The second remark is related to the biological relevance of our results. In our study, we have focused on recombination-driven situation and ignored mutation. In evolution of prokaryotes, there are many situations where recombination rate is much larger than mutation rate. In fact, we can see spontaneous horizontal gene transfer rate in some prokaryotes is larger than mutation rate in [20]. Furthermore, there is another evidence that evolution is mainly driven by horizontal gene transfer in some prokaryotes, as reported in [21–24]. Since we consider the situation where selection is stronger than recombination, the result is applicable mainly to prokaryotes, and the applicability to sexually-reproducing eukaryotes with larger recombination rate may be limited. Note that mutation in realistic systems is almost deleterious, and the rate of mutation flipping genes as in our model is expected to be further small. Therefore, when population size is relatively small, a phenomenon reported in this paper may be observed in some prokaryotes.

Third, our study might look similar to a study by Barton [25], in that both investigate the dynamics of introgression in the presence of linkage disequilibrium. However, our study differs from that of Barton in the following aspect: whereas Barton’s study considers the introgression of deleterious alleles linked to each other, our study considers the introgression of beneficial alleles linked to deleterious alleles. Our study also differs in the conclusion about how introgression depends on the strength of selection. Barton’s study shows that the introgression of deleterious alleles is a monotonic function of the selection coefficient. In contrast, our study shows that the introgression of beneficial alleles is a non-monotonic function of the selection coefficient, the result essentially due to the interactions between beneficial and deleterious alleles through linkage disequilibrium.

Fourth, previous studies find that an optimal recombination rate exists in evolution driven by mutation and recombination [26, 27]. One might think that our result on the optimal value in selection pressure can be trivially deduced from their results. However, we believe that this is not the case because in our case the optimal value of *s* is almost independent of *r*, as seen from Eq (6). In addition, an optimal recombination rate in [26] may come from the loss of genetic diversity due to copy-and-paste-type recombination, which differs from recombination considered in our models (see Eq (4)). The paper [27] reports an optimal recombination rate in a state in which fitness is stationary over time, whereas we here focus on a state in which fitness steadily increases over time. Therefore, we think that the mechanism by which an optimal *s* value arises investigated in our work differs from those by which optimal recombination rates arise investigated in the previous studies.

Fifth, evolution can slow down even in a mutation-driven situation when a fitness landscape is rugged and population is finite; however, this differs from our result. When selection pressure is too strong, whole population gets stuck into a local maximum of a rugged fitness landscape, and the speed of evolution becomes small. Although this mechanism has not been studied systematically, the population-size dependence of the speed of evolution on rugged fitness landscapes has recently attracted much attention [28–31]. However, whereas these phenomena result from the ruggedness of fitness landscapes, those reported in this paper come from the decrease of genetic variation due to selection. Therefore, we believe that the two are different phenomena.

We finally remark that the phenomenon reported in this paper may be similar to negative differential resistance (NDR) [32–38]. NDR is a phenomenon in which particle current becomes smaller by increasing external force. NDR has been observed in many physical systems, and is regarded as a common property of transport in crowded environments. In our paper, we find a phenomenon in which the rate of evolution becomes smaller by increasing selection pressure. The fact that recombination does not work as population becomes homogeneous seems to be similar to the fact that particles cannot move as the positions of particles become close to each other in kinetically constrained models (KCM) [39], which are one of the models for glass with smooth energy landscapes and exhibit NDR. In this analogy, selection pressure corresponds to external force for KCM [35], and the fact that fitness cannot increase as genotypes become homogeneous corresponds to the fact the particles cannot flow in the direction of external force as particles get crowded. Therefore, while rugged fitness landscape models are similar to spin glass models [40], our model may be similar to KCM. Further similarity to KCM will be studied in future.

## References

[1] Hartl DL, Clark AG. Principles of population genetics. Sinauer Associates: Sunderland, MA; 1997.

[2] Tsimring LS, Levine H, Kessler DA. RNA Virus Evolution via a Fitness-Space Model. Phys. Rev. Lett. 1996 76, 4440 10.1103/PhysRevLett.76.4440 10061290

[3] Kessler DA, Levine H, Ridgway D, Tsimring L. Evolution on a Smooth Landscape. J. Stat. Phys. 1997 87, 519 10.1007/BF02181235

[4] Gillespie JH. Population genetics: a concise guide. Johns Hopkins University Press: Baltimore, MD; 1998.

[5] Gerrish PJ, Lenski RE. The fate of competing beneficial mutations in an asexual population. Genetica 1998 102/103, 127 10.1023/A:1017067816551 9720276

[6] Wilke CO. The Speed of Adaptation in Large Asexual Populations. Genetics 2004 167, 2045 10.1534/genetics.104.027136 15342539 PMC1470994

[7] Desai MM, Fisher DS. Beneficial Mutation-Selection Balance and the Effect of Linkage on Positive Selection. Genetics 2007 176, 1759 10.1534/genetics.106.067678 17483432 PMC1931526

[8] Park SC, Krug J. Clonal interference in large populations. Proc. Natl. Acad. Sci. USA 2007 104, 18135 10.1073/pnas.0705778104 17984061 PMC2084309

[9] Good BH, Rouzine IE, Balick DJ, Hallatschek O, Desai MM. Distribution of fixed beneficial mutations and the rate of adaptation in asexual populations. Proc. Natl. Acad. Sci. USA 2012 109, 4950 10.1073/pnas.1119910109 22371564 PMC3323973

[10] Maynard-Smith J. The Evolution of Sex. Cambridge University Press: Cambridge; 1978.

[11] Neher RA, Shraiman BI, Fisher DS. Rate of Adaptation in Large Sexual Populations. Genetics 2010 184, 467 10.1534/genetics.109.109009 19948891 PMC2828726

[12] Watson RA, Weinreich DM, Wakeley J. Genome structure and the benefit of sex. Evolution 2011 65, 523 10.1111/j.1558-5646.2010.01144.x 21029076

[13] Ochman H, Lawrence JG, Groisman EA. Lateral gene transfer and the nature of bacterial innovation. Nature 2000 405, 299 10.1038/35012500 10830951

[14] Koonin EV, Makarova KS, Aravind L. Horizontal gene transfer in prokaryotes: quantification and classification. Annu. Rev. Microbiol. 2001 55, 709 10.1146/annurev.micro.55.1.709 11544372 PMC4781227

[15] Takeuchi N, Kaneko K, Koonin EV. Horizontal gene transfer can rescue prokaryotes from Muller’s ratchet: benefit of DNA from dead cells and population subdivision. G3: Genes, Genomes, Genetics 2014 4, 325 10.1534/g3.113.009845 24347631 PMC3931566

[16] Takeuchi N, Cordero OX, Koonin EV, Kaneko K. Gene-specific selective sweeps in bacteria and archaea caused by negative frequency-dependent selection. BMC Biology 2015 13, 20 10.1186/s12915-015-0131-7 25928466 PMC4410459

[17] Niehus R, Mitri S, Fletcher AG, Foster KR. Migration and horizontal gene transfer divide microbial genomes into multiple niches. Nat. Commun. 2015 6, 8924 10.1038/ncomms9924 26592443 PMC4673824

[18] Dixit P, Pang TY, Maslov S. Recombination-driven genome evolution and stability of bacterial species. Genetics 2017 10.1534/genetics.117.300061 28751420 PMC5586378

[19] Peliti L. Introduction to the statistical theory of Darwinian evolution. arXiv 1997 cond-mat/9712027.

[20] Overballe-Petersen S, Harms K, Orlando LAA, Moreno-Mayar JV, Rasmussen S, Dahl TW, et al Bacterial natural transformation by highly fragmented and damaged DNA. Proc. Natl. Acad. Sci. USA 2013 110, 19860 10.1073/pnas.1315278110 24248361 PMC3856829

[21] Feil EJ, Smith JM, Enright MC, Spratt BG. Estimating Recombinational Parameters in Streptococcus pneumoniae From Multilocus Sequence Typing Data. Genetics 2000 154, 1439 10747043 10.1093/genetics/154.4.1439 PMC1461021

[22] Vos M, Didelot X. A comparison of homologous recombination rates in bacteria and archaea. The ISME Journal 2009 3, 199 10.1038/ismej.2008.93 18830278

[23] Puigbò P, Lobkovsky AE, Kristensen DM, Wolf YI, Koonin EV. Genomes in turmoil: quantification of genome dynamics in prokaryote supergenomes. BMC Biology 2014 12, 66 10.1186/s12915-014-0066-4 25141959 PMC4166000

[24] Lin M, Kussell E. Correlated Mutations and Homologous Recombination Within Bacterial Populations. Genetics 2017 205, 891 10.1534/genetics.116.189621 28007887 PMC5289858

[25] Barton NH. Multilocus clines. Evolution 1983 37, 454 10.1111/j.1558-5646.1983.tb05563.x 28563316

[26] Cohen E, Kessler DA, Levine H. Recombination Dramatically Speeds Up Evolution of Finite Populations. Phys. Rev. Lett. 2005 94, 098102 10.1103/PhysRevLett.94.098102 15784005

[27] Lobkovsky AE, Wolf YI, Koonin EV. Evolvability of an Optimal Recombination Rate. Genome Biol. Evol. 2016 8, 70 10.1093/gbe/evv249 PMC4758245 26660159

[28] Rozen DE, Habets MGJL, Handel A, de Visser JAGM. Heterogeneous Adaptive Trajectories of Small Populations on Complex Fitness Landscapes. PLoS ONE 2008 3, e1715 10.1371/journal.pone.0001715 18320036 PMC2248617

[29] Handel A, Rozen DE. The impact of population size on the evolution of asexual microbes on smooth versus rugged fitness landscapes. BMC Evolutionary Biology 2009 9, 236 10.1186/1471-2148-9-236 19765292 PMC2753573

[30] Jain K, Krug J, Park SC. Evolutionary advantage of small populations on complex fitness landscapes. Evolution 2011 65, 1945 10.1111/j.1558-5646.2011.01280.x 21729050

[31] Ochs IE, Desai MM. The competition between simple and complex evolutionary trajectories in asexual populations. BMC Evolutionary Biology 2015 15, 55 10.1186/s12862-015-0334-0 25881244 PMC4391547

[32] Slater GW, Guo HL, Nixon GI. Bidirectional Transport of Polyelectrolytes Using Self-Modulating Entropic Ratchets. Phys. Rev. Lett. 1997 78, 1170 10.1103/PhysRevLett.78.1170

[33] Zia RKP, Praestgaard EL, Mouritsen OG. Getting more from pushing less: Negative specific heat and conductivity in nonequilibrium steady states. Am. J. Phys. 2002 70, 384 10.1119/1.1427088

[34] Kostur M, Machura L, Hänggi P, Luczka J, Talkner P. Forcing inertial Brownian motors: Efficiency and negative differential mobility. Physica A 2006 371, 20 10.1016/j.physa.2006.04.086

[35] Sellitto M. Asymmetric Exclusion Processes with Constrained Dynamics. Phys. Rev. Lett. 2008 101, 048301 10.1103/PhysRevLett.101.048301 18764369

[36] Baerts P, Basu U, Maes C, Safaverdi S. Frenetic origin of negative differential response. Phys. Rev. E 2013 88, 052109 10.1103/PhysRevE.88.052109 24329216

[37] Bénichou O, Illien P, Oshanin G, Sarracino A, Voituriez R. Microscopic Theory for Negative Differential Mobility in Crowded Environments. Phys. Rev. Lett. 2014 113, 268002 10.1103/PhysRevLett.113.268002 25615388

[38] Baiesi B, Stella AL, Vanderzande C. Role of trapping and crowding as sources of negative differential mobility. Phys. Rev. E 2015 92, 042121 10.1103/PhysRevE.92.042121 26565182

[39] Ritort F, Sollich P. Glassy dynamics of kinetically constrained models. Adv. Phys. 2003 52, 219 10.1080/0001873031000093582

[40] Mézard M, Parisi G, Virasoro MA. Spin glass theory and beyond. World Scientific: Singapore; 1987

